# Mortality on extracorporeal membrane oxygenation: Evaluation of independent risk factors and causes of death during venoarterial and venovenous support

**DOI:** 10.1177/02676591231212997

**Published:** 2023-11-07

**Authors:** Johannes Deinzer, Alois Philipp, Lukasz Kmiec, Jing Li, Sigrid Wiesner, Sebastian Blecha, Walter Petermichl, Matthias Lubnow, Daniele Camboni, Christof Schmid, Andrea Stadlbauer

**Affiliations:** 1 Department of Internal Medicine, 39070 University Medical Center Regensburg, Regensburg, Germany; 2 Department of Cardiothoracic Surgery, 39070 University Medical Center Regensburg, Regensburg, Germany; 3 Department of Anaesthesiology, 39070 University Medical Center Regensburg, Regensburg, Germany

**Keywords:** cardiogenic shock, cause of death, critical care, extracorporeal membrane oxygenation, risk factors

## Abstract

**Introduction:**

Most patients on extracorporeal membrane oxygenation (ECMO) decease during therapy on the system. However, the actual causes of death have not been studied sufficiently. This study analyses the etiology, prevalence, and risk factors for the outcome variable death during ongoing ECMO for all patients and divided according to venoarterial (VA) or venovenous (VV) support.

**Methods:**

We retrospectively analysed all patients receiving ECMO support at our institution between March 2006 to January 2021. Only the patients deceased during ongoing support were included.

**Results:**

2016 patients were placed on VA (*n* = 1168; 58%) or VV (*n* = 848; 42%) ECMO; 759 patients (37.7%) deceased on support. The causes of death differed between the support types: VA ECMO patients mostly died from cerebral ischemia (34%), low-cardiac output (LCO; 24.1%) and multi-organ failure (MOF; 21.6%), whereas in VV ECMO cases, refractory respiratory failure (28.2%), and sepsis (20.4%) dominated. Multivariate regression analysis revealed cardiopulmonary resuscitation (CPR) and acidosis prior to ECMO as risk factors for dying on VA ECMO, while high inotropic doses pre-ECMO, a high fraction of inspired oxygen on day 1, elevated lactate dehydrogenase, and international normalized ratio levels lead to an unfavourable outcome in VV ECMO patients.

**Conclusion:**

Even in highly experienced centers, ECMO mortality remains high and occurs mainly on support or 24 h after its termination. The causes of death differ between VV and VA ECMO, depending on the underlying diseases responsible for the need of extracorporeal support.

## Introduction

Over the last decades, extracorporeal membrane oxygenation (ECMO) has become an indispensable constituent in the treatment of severe cardiac and pulmonary failure in intensive care and emergency medicine. Though technical aspects and the management of patients on ECMO are constantly improving, not least because of the recent Covid pandemic, mortality in patients with extracorporeal support remains high, ranging from 29 to 43% in veno-venous (VV) ECMO and 40–84% in veno-arterial (VA) ECMO cases.^1,2^

Several studies address the mortality of ECMO, which seems to be highest 48 h after implantation, but until today, only few studies focus on the causes of death during ongoing ECMO support or the factors predisposing patients to death while still supported.^3,4^

Therefore, the aim of this study was to analyse the etiology, prevalence, and risk factors for death during ongoing ECMO support for all patients and to stratify them according to the support type (VA vs VV ECMO).

## Methods

### Study design and patient selection

A retrospective analysis of our prospective institutional ECMO database between March 2006 and January 2021 was performed. All patients deceased on ECMO with either VA or VV support were included in this study. Patients converted to V-AV ECMO were excluded from this study. If conversion of ECMO modality was necessary, the last modality was used for defining ECMO mode in this study.

Approval for this study was obtained from the ethics board of Regensburg University on March 24, 2021 (Study title: Mortality on extracorporeal life support: evaluation of independent risk factors and causes of death; Case number: 21-2256-104). Individual patient approval was waived due to the retrospective nature of the study.

The procedures followed were in accordance with the ethical standards of the responsible committee on human experimentation (institutional or regional) and with the Helsinki Declaration of 1975.

### Definition of outcome variables

Death on ECMO support was defined as death during ongoing ECMO support or death within 24 h after its termination.

ECMO support was terminated in patients with irreversible brain damage or patients with terminal heart or lung failure without further therapeutic option, as well as refractory multi-organ failure after thorough consideration and joint decision making with the next of kin, knowing that death was to be expected.

The following most common causes of death were included into the independent risk factor analysis: bleeding, cerebral bleeding, cerebral ischemia, intestinal bleeding, low cardiac output (LCO), multi-organ failure (MOF), pulmonary embolism, respiratory failure, and sepsis.

Bleeding was defined as bleeding from cannulation site, bleeding from operation site or every other bleeding complication except cerebral or gastrointestinal bleeding requiring more than three packed red blood cell (RBC) transfusions within 24 h.

Cerebral ischemia or cerebral bleeding was defined as irreversible cessation of all brain functions or major neurological insults with grave prognosis caused by either ischemia or bleeding, clinically evaluated by a neurologist with brain death assessment or proven by electroencephalography, followed by the withdrawal of care.

Intestinal bleeding was defined as severe bleeding from the gastrointestinal tract necessitating transfusion of more than three RBC within 24 h.

LCO was defined as persistent cardiogenic shock despite the use of inotropic agents or mechanical circulatory support without weaning possibility and no other therapeutic option (e.g. long-term mechanical circulatory device, heart transplantation) remaining.

MOF was defined as irreversible failure of more than two organ systems and a sequential organ failure assessment score (SOFA) above nine points leading to death.

Death due to pulmonary embolism was defined as obstruction in the pulmonary artery due to a clot, tumor, air, or fat resulting in persistent, irreversible cardiogenic shock.

Persistent respiratory failure was defined as a Horowitz Index under 100 mmHg,^5,6^ the need for aggressive ventilation parameters (PEEP >5 cm H_2_O) with irreversible lung damage and no further therapeutic option remaining.

Death following sepsis was defined as persistent septic shock caused by infection with systolic blood pressure under 90 mmHg despite adequate fluid substitution, vasopressors and ECMO support leading to terminal end-organ hypoperfusion.

### ECMO management

In-hospital implementation of ECMO was performed by a team of trained doctors, perfusionists, and nurses, and managed according to our standardized institutional protocol, which has been previously described for VA and VV ECMO.^7,8^ ECMO-assisted cardiopulmonary resuscitation (ECPR) was provided by our mobile ECMO team, which undertakes ECMO implantation in external hospitals, doctors’ offices, and even out of hospital at scenes of accidents.

The criteria for ECMO therapy at our institution are liberal. However, we do not implement ECMO in patients with a cardiopulmonary resuscitation (CPR) duration of less than 15 min, known irreversible brain damage, terminal malignancy, traumatic injury with uncontrolled bleeding, unwitnessed circulatory arrest, and an existing, credible declaration that the patient does not wish to receive life-prolonging measures such as mechanical circulatory assist devices. In patients aged over 70 years, decisions are made on an individual basis.

Mechanical ventilation was initiated according to the institution’s standard protocol, and included an open-lung strategy with protective lung settings according to published guidelines.^9^

### Statistical analysis

Statistical analysis was performed with IBM SPSS Statistics 25 (IBM, New York, USA). For tabulated data collection before import into SPSS we used Excel for Windows (Microsoft Corporation, Redmond, WA, USA).

Continuous data was presented as mean with standard deviation or median with interquartile range (IQR). Normal distribution was formally tested with the Shapiro-Wilks-test. Categorical data was listed as frequencies and percentages. Comparison of continuous variables was performed using the student’s *t*-test and the Mann-Whitney U test. Categorical variables were compared using the chi-square test. A *p*-value under .05 was considered statistically significant. After univariate analysis, a stepwise multivariate logistic regression analysis was performed to identify independent risk factors for death on ECMO and the different causes of death.

Missing data was listwise deleted.

## Results

### Study population

From March 2006 to January 2021, 2016 patients were placed on ECMO (VA *n* = 1168; 58%; VV *n* = 848; 42%). Conversion of therapy took place in 36 of VA ECMO patients (16 to VV-ECMO and 20 to VA-V ECMO) and 12 of VV ECMO patients (9 to VA-ECMO, 3 to VA-V ECMO).

272 (13.5%) patients died after a mean duration of 17.9 ± 21 days after weaning from ECMO.

985 (48.9%) patients survived till discharge after a mean hospital stay of 40.7 ± 51 days.

759 (37.6%) patients died while still on ECMO support or within 24 h after its withdrawal. In 44.8% (*n* = 34) of these deceased patients, decision was made together with the patient’s family to terminate ECMO therapy ultimately leading to death within 24 h after ECMO termination: Most of these patients had irreversible brain damage (*n* = 16; 47%), seven (20.6%) died from refractory LCO without further therapeutic option, six (17.6%) from MOF, two patients (5.9%) died from septic shock and bleeding and one patient suffered refractory respiratory failure (2.9%). Characteristics of the 759 deceased patients are summarized in Table 1.

**Table 1.** Demographic data of patients dying on support.

	Total (*n* = 759)	VA ECMO (*n* = 514)	VV-ECMO (*n* = 245)	*p*-value
Age (years)	56.4 ± 15.4	58.3 ± 14.3	52.5 ± 14.5	**<.001**
Male	532 (70.1%)	359 (69.8%)	173 (70.6%)	.625
Height (cm)	170.7 ± 11.5	171 ± 9.6	171 ± 8.8	.894
Weight (kg)	84.4 ± 22.5	84 ± 21	94.5 ± 28	.194
BMI (kg/m^2^)	29.1 ± 10.1	29.2 ± 16	32.3 ± 10	.108
ECMO support (days)	7.5 ± 12.5	4.2 ± 8.3	14.4 ± 16.5	**<.001**
ICU stay (days)	13.2 ± 24.3	9.5 ± 20	21 ± 30	**<.001**
Post-cardiac surgery	149 (19.6%)	149 (28.9%)	0	—
Pre-lactate (mmol/l)	9.5 ± 6.9	12.2 ± 6.3	4.3 ± 5	**<.001**
Pre-norepinephrine (µg/kg/min)	0.5 ± 0.7	0.49 ± 0.73	0.5 ± 0.8	.489
Pre-epinephrine (µg/kg/min)	0.2 ± 0.3	0.21 ± 0.28	0.12 ± 0.01	**<.001**
Pre-MAP (mmHg)	55.2 ± 17.3	48 ± 15	58 ± 12.5	**<.001**
Pre-pH	7.2 ± 0.2	7.2 ± 0.15	7.05 ± 0.2	**.002**
Pre-prothrombin time (%)	60.1 ± 27.1	55 ± 28	70.5 ± 22.7	**<.001**
Pre-LDH (U/l)	1050 ± 1974	1129 ± 2136	895 ± 1600	.759
Pre-d-dimer (mg/l)	16.1 ± 13.8	16.2 ± 14.2	15.8 ± 14.3	.889
Peak NSE-level (µg/l)	146 ± 171	168 ± 183	80.3 ± 105	**<.001**
Peak fHb (mg/l)	499 ± 554	547 ± 579	388 ± 493	**<.001**
Pre-implant CPR	431 (56.8%)	395 (76.8%)	36 (14.7%)	**<.001**

Notes: mean (±SD); BMI: body mass index, ICU: intensive care unit, fHb: free hemoglobin levels, MAP: mean arterial pressure, LDH: lactate dehydrogenase, NSE: neuronspecific enolase. Bold represents the significant *p*-values.

The table presents the demographic data of all deceased patients, outlining the differences between VA and VV ECMO patients with statistical significance.

Most of the deceased patients were male (*n* = 532, 70.1%). The median age of the study cohort was 58.3 years (IQR: 47.8–67.8 years).

The median ECMO duration was 3 days (IQR: 1–9 days), median intensive care unit (ICU) stay until death was 5 days (IQR: 2–14 days); both parameters were significantly higher in VV ECMO patients (*p* < .001).

Most deaths occurred in the VA ECMO group (VA: 514 patients, 67.7%; VV: 245 patients, 32.3%).

Approximately one third of the VA ECMO patients (*n* = 149; 29%) underwent cardiac surgery prior to the established ECMO therapy.

CPR on ECMO support was necessary in 576 of the patients (75.9%), with a significantly higher proportion in the VA-group (VA ECMO: 71.2% (410) versus VV ECMO: 28.8% (166); *p* < .001).

The indications for VA ECMO were VA ECMO-assisted CPR (ECPR; proportion of resuscitated out of hospital (OHCA): 64/295 (21.7%)) in 79.8% (295/410); 16.8% (69/410) were successfully resuscitated but showed symptoms of persistent cardiogenic shock (CS) and received VA ECMO less than 12 h after CPR. 12.5% (64/514) could not be weaned from cardiopulmonary bypass during cardiac surgery and 16.9% (87/514) received VA ECMO due to LCO.

The indication for VV-ECMO was ARDS, which was either caused directly (e.g. pneumonia, aspiration, lung contusion) in 62.4% (*n* = 153), or indirectly (e.g. surgery related, drug-induced) in 37.6% (*n* = 92).

### Predictors for death on ECMO

#### VA ECMO

Multiple variables were found to be predictive for death during ongoing support in VA ECMO patients in univariate analysis (Table 2): ECPR (OR 2.13; 95% CI: 1.68–2.69; *p* < .001) or general CPR prior to ECMO (OR 6.8; 95% CI: 5.2–8.9; *p* < .001), as well as OHCA (OR 4.7; 95% CI: 2.98–7.3; *p* < .001) elevated the risk significantly. A low BMI (OR 0.97; 95% CI: 0.95–0.99; *p* < .001), low MAP prior to implantation (OR 0.95; 95% CI: 0.95–0.96; *p* < .001) were also predictive for dying on support, as well as the need for inotropic agents prior to ECMO (OR 6.9; 95% CI: 3.9–12.2; *p* < .001), acidosis (OR 0.16; 95% CI: 0.07–0.38; *p* < .001) and elevated lactate levels prior to ECMO (OR 1.013; 95% CI: 1.011–1.016; *p* < .001).

**Table 2.** Univariate regression analysis for predictors of death for VA ECMO patients.

	OR	95% CI	*p*-value
Pre-CPR	6.8	5.2–8.9	**<.001**
ECPR	2.125	1.68–2.69	**<.001**
pH	0.16	0.07–0.38	**<.001**
Pre-epinephrine	6.9	3.9–12.2	**<.001**
MAP	0.95	0.946–0.96	**<.001**
BMI	0.97	0.95–0.99	**<.001**
OHCA	4.7	2.9–7.3	**<.001**
Pre-lactate	1.013	1.01–1.016	**<.001**
Pre-prothrombin time	0.985	0.98–0.99	**<.001**
Pre-aPTT	1.009	1.005–1.01	**<.001**
Pre-fibrinogen	0.996	0.995–0.997	**<.001**

Notes: CPR: cardiopulmonary resuscitation; ECPR: ECMO-assisted cardiopulmonary resuscitation; MAP: mean arterial pressure; BMI: body mass index; OHCA: out of hospital cardiac arrest; aPTT: activated partial thromboplastin time. Bold represents the significant *p*-values.

The table depicts the factors predisposing for death on VA ECMO patients.

Signs for a deranged coagulation system, like low prothrombin time (OR 0.99; 95% CI: 0.98–0.99; *p* < .001), elevated activated partial throboplastin time (OR 1.01; 95% CI: 1.005–1.012; *p* < .001) or low fibrinogen levels prior to implantation (OR 0.996; 95% CI: 0.995–0.997; *p* < .001) had a negative impact on survival.

However, when analyzing these variables in the multivariate analysis, only CPR prior to ECMO (OR 2.02; 95% CI: 1.03–3.96; *p* = .04) and acidosis prior to ECMO (OR 0.11; 95% CI: 0.01–0.89; *p* = .04) remained significant for dying during VA ECMO support.

#### VV ECMO

Similarly to the VA ECMO patients, univariate regression analysis in VV ECMO patients proved CPR prior to ECMO (OR 7.7; 95% CI: 5.34–10.33; *p* < .001), elevated lactate levels (OR 1.021; 95% CI: 1.017–1.025; *p* < .001), acidosis (OR 0.104; 95% CI: 0.03–0.33; *p* < .001), a low MAP prior to ECMO (OR 0.96; 95% CI: 0.95–0.97; *p* < .001) and the need for inotropic agents (OR 11.1; 95% CI: 5.24–23.6; *p* < .001) to be predictive for death during support, as well as elevated aPTT (OR 1.018; 95% CI: 1.01–1.03; *p* < .001) and low prothrombin time (OR 0.98; 95% CI: 0.97–0.984; *p* < .001). But unlike the VA ECMO patients, VV ECMO patients also died with a higher probability if they were older (OR 1.012; 95% CI: 1.002–1.021; *p* = .017), in need of high doses of vasopressors prior to ECMO initiation (OR 1.03; 95% CI: 1.05–1.59; *p* = .02) and a high fraction of inspired oxygen (FiO_2_) (OR 3.5; 95% CI: 2.13–5.43; *p* < .001). Elevated lactate dehydrogenase levels (LDH) (OR 1.002; 95% CI: 1.001–1.003; *p* < .001), elevated INR prior to ECMO (OR 1.63; 95% CI: 1.34–1.98; *p* < .001) and on day one (OR 2.12; 95% CI: 1.6–2.81; *p* < .001), low fibrinogen (OR 0.996; 95% CI: 0.995–0.997; *p* < .001) and low platelet counts (OR 0.997; 95% CI: 0.996–0.999; *p* = .003) were predictive for death on VV ECMO, too.

The detailed univariate regression analysis is displayed in Table 3.

**Table 3.** Univariate regression analysis for predictors of death for VV ECMO patients.

	OR	95% CI	*p*-value
Age	1.01	1.002–1.02	**.017**
CPR	7.4	5.34–10.33	**<.001**
pH	0.10	0.03–0.33	**<.001**
Pre-norepinephrine	1.03	1.05–1.59	**.018**
Pre-epinephrine	11.1	5.24–23.6	**<.001**
MAP	0.96	0.95–0.97	**<.001**
FiO2 day 1	3.5	2.13–5.43	**<.001**
Pre-LDH	1.002	1.001–1.003	**<.001**
Pre-lactate	1.02	1.017–1.03	**<.001**
Pre-prothrombin time	0.98	0.97–0.98	**<.001**
Pre-aPTT	1.018	1.013–1.025	**<.001**
Pre-INR	1.6	1.34–1.98	**<.001**
INR day 1	2.12	1.6–2.81	**<.001**
Pre-fibrinogen	0.996	0.995–0.997	**<.001**
Pre-thrombocytes	0.997	0.996–0.999	**.003**

Notes: CPR: cardiopulmonary resuscitation; MAP: mean arterial pressure; FiO2: fraction of inspired oxygen; LDH: lactate dehydrogenase; aPTT: activated partial thromboplastin time; INR: international normalized ratio. Bold represents the significant *p*-values.

The table depicts the factors predisposing for death on VV ECMO patients.

After applying these factors to multivariate regression analysis, the following remained significant: high doses of inotropic agents (OR 5.2; 95% CI: 2.3–11.1; *p* = .03), a high FiO_2_ 1 day after ECMO initiation (OR 9.3; 95% CI: 2.3–37.8; *p* = .02), elevated LDH prior to ECMO (OR 1.004; 95% CI: 1.002–1.01; *p* = .05), as well as elevated international normalized ratio (INR) 1 day after initiation (OR 3.9; 95% CI: 2.5–41.4; *p* = .008).

### Causes of death on ECMO

The underlying causes of death on ECMO were as follows: bleeding, cerebral bleeding, cerebral ischemia, intestinal bleeding, LCO, MOF, pulmonary embolism, respiratory failure, and sepsis.

**Figure 1.**
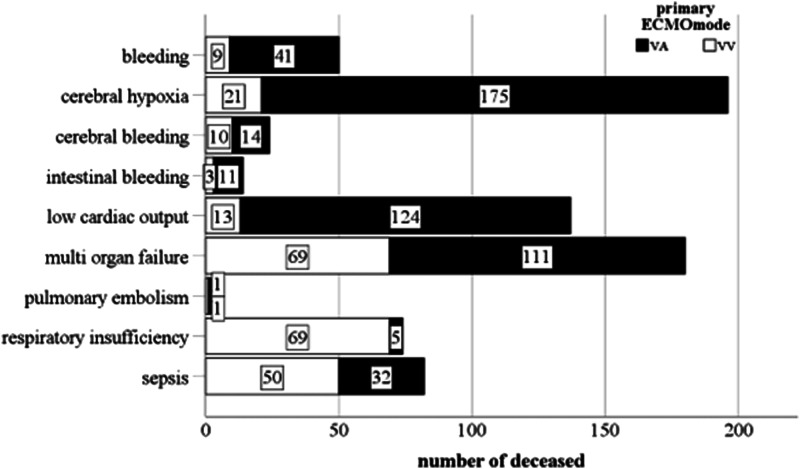
Distribution of causes of death on ECMO according to support type in our patient cohort.

The main causes of death during extracorporeal circulatory support were cerebral ischemia (*n* = 185; 24.4%) and MOF (*n* = 174; 22.9%). The distribution of the other causes of death is depicted in Figure 1.

In comparison, there was no significant difference in causes of death between patients dying on ECMO support and those who died 24 h after ECMO weaning.

VA ECMO patients died significantly more often from bleeding (8% vs 4%; *p* = .025), cerebral ischemia (34% vs 8.6%; *p* < .001), LCO (24.1% vs 5.3%; *p* < .001) and MOF (28.2% vs 21.6%; *p* = .05), while VV ECMO patients mostly died from respiratory failure (28.2% vs 1%; *p* < .001) and sepsis (20.4% vs 6%; *p* < .001).

When regarding the different indications for VA support, we found LCO (39.6%) and MOF (33.6%) to be the most common causes of death in postcardiotomy patients.

Patients who received ECPR mostly died due to cerebral ischemia (45.3%) or LCO (22.3%), while patients who were put on ECMO due to LCO mostly died from cerebral ischemia (47.7%) and MOF (41.9%).

### Multivariate regression analysis for predictors for the most common causes of death on ECMO

After analysing the predictors for death during ECMO support in general, we evaluated the factors predisposing for the most common causes of death each individually: bleeding, cerebral ischemia, LCO, MOF, respiratory failure, and sepsis.

The detailed regression analysis can be found in Table 4.

**Table 4.** Multivariate regression analysis for risk factors for the different causes of death during ECMO support.

	Bleeding	Cerebral ischemia	LCO	MOF	Respiratory failure	Sepsis
aPTT	—	—	—	—	—	1.03 (1.02–1.04); *p* < .001
CPR	—	—	4.5 (1.6–12.7); *p* = .004	—	—	16.6 (5.8–47.7); *p* < .001
ECPR	—	65.4 (6.2–690.7); *p* < .001	—	—	—	—
Pre-LDH	—	1.001 (1–1.002); *p* = .013	—	—	—	—
MAP	—	—	0.97 (0.93–1.001); *p* = .05	—	—	—
Peak-NSE levels	—	1.006 (1–1.012); *p* = .05	—	—	—	—
PAW	—	—	—	—	1.32 (1.07–1.62); *p* = .01	—
Previous cardiac surgery	—	—	3.5 (1.24–9.9); *p* = .02	3.3 (1.02–10.6); *p* < .05	—	—
PT	0.99 (0.97–0.99); *p* = .033	—	—	—	—	—
Support duration	—	—	—	—	1.15 (1.07—1.24); *p* < .001	—
VA ECMO	2.65 (1.16–6.05); *p* = .020	—	—	—	—	—

aPTT: activated partial thromboplastin time; CPR: cardiopulmonary resuscitation; ECPR: ECMO-assisted cardiopulmonary resuscitation; IL-2: interleukin-2; LDH: lactate dehydrogenase; MAP: mean arterial pressure; NSE: neuron-specific enolase; PAW: peak airway pressure; PT: prothrombin time; VA ECMO: veno-arterial extracorporeal membrane oxygenation.

The table depicts the risk factors leading to the different most common causes of death: bleeding, cerebral ischemia, LCO, MOF, respiratory failure and sepsis.

Dying from bleeding complications during support was more common in VA ECMO patients and patients with increased prothrombin time prior to ECMO initiation.

Patients who were resuscitated (ECPR), who had elevated LDH levels and higher peak NSE levels, showed a higher risk for dying of cerebral ischemia, while higher MAP before ECMO initiation seemed to have a protective effect.

LCO as cause of death was more common in resuscitated patients with low MAP prior to ECMO, as well as in postcardiotomy patients. When examining the VA ECMO patients only, old age was a risk factor for dying of LCO, too (OR 1.04; 95%-CI 1.002–1.08; *p* = .04).

Postcardiotomy ECMO was predictive for dying of MOF, as well. Additionally, VA ECMO patients dying from MOF had a longer support duration (OR 1.11; 95%-CI 1.01–1.23; *p* = .03).

Respiratory failure as cause of death was more likely in patients with a long support duration and an elevated peak airway pressure pre-ECMO. VV ECMO patients with longer support duration (OR 1.13; 95%-CI: 1.07–1.20; *p* < .001) and CPR (OR 17.9; 95%-CI: 2.41–134.13; *p* = .005) had a higher risk for dying of respiratory failure. Although elevated PEEP and peak pressure levels seemed to be influential in VA ECMO patients in univariate analysis, none of the parameters could be confirmed in multivariate analysis.

Previous CPR and elevated aPTT levels ahead of ECMO made dying from sepsis more likely especially in VV ECMO patients (CPR: OR 5.3; 95%-CI: 1.5–18.9; *p* = .01; aPTT: OR 1.02; 95%-CI: 1.01–1.31; *p* = .008).

## Discussion

In this single-centre study comprising 2016 ECMO runs during 15 years of our institutional experience, we analysed the causes of death during ECMO support for all patients and stratified according to the support type. Moreover, we evaluated clinical risk factors for death during ECMO support or within 24 h after withdrawal and for the different causes of death.

The major causes of death during ongoing ECMO were cerebral ischemia and MOF. Stratified according to support type, causes of death on VA ECMO were mainly cerebral ischemia, LCO and MOF, while VV ECMO patients mostly died from refractory respiratory failure and sepsis.

We found CPR and acidosis pre-ECMO to be predictive for dying on support in VA ECMO patients, whereas the need for high inotropic doses, high FiO_2_ on day one, elevated LDH and INR levels were predictive for mortality in VV ECMO patients.

With a proportion of 34% cerebral ischemia was the leading cause of death in our VA ECMO group, which is easily be explained by the high proportion of ECPR patients (79.8%) in this group. This is further supported by findings by Lorusso et al., who already established an association between CPR and adverse neurological outcome leading to a higher morbidity and mortality.^10^

Postcardiotomy cardiogenic shock is a highly fatal condition itself and even if treated with ECMO, mortality in these patients remains high.^11,12^ Nearly one third of our VA ECMO patients received ECMO following cardiotomy. Weaning failure in refractory LCO in patients who are not suitable for further therapeutic measurements (e.g. long-term mechanical circulatory devices, heart transplantation) ultimately leads to withdrawal of care. Provaznik and Khorsandi et al. also determined LCO as the leading cause of death in postcardiotomy ECMO patients.^13,14^

While our study is the first to analyse the specific causes of death during ongoing ECMO support in VA ECMO patients, there already exist studies concerning the causes of death during ongoing VV ECMO support.

Concordant with previous investigations by our group, refractory respiratory failure with no option for lung transplantation and sepsis were the leading causes of death in our VV ECMO group. In contrast, in their meta-analysis, Heuts et al. reported that multiorgan failure, closely followed by hemorrhage and sepsis, were the most common causes of death during VV ECMO.^15,16^ Half of our patient population received VV ECMO due to bacterial or viral pneumonia, which might explain the high proportion of patients dying from septic shock.

Several studies investigating predictors for in-hospital death, death after weaning or mortality during follow up exist for VA and VV ECMO.^13,14,17,18^

Our study aimed to identify predictors for the outcome variable death during ongoing support.

Still, many of our findings coincide with previously known risk factors for overall mortality:

As already mentioned above, postcardiotomy cardiogenic shock is a well-known factor influencing survival, like previous CPR or CPR during ECMO support.^11,19^

We found acidosis at initiation of therapy to be predictive for mortality on VA ECMO; these results fit in with the current literature, where these parameters are also part of the PREDICT VA ECMO score, a prognostic model for survival developed by Wengenmayer et al.^20^

High doses of vasopressors and inotropes suggest a severe deterioration in the hemodynamics of VA and VV ECMO patients, causing hypoperfusion of end-organs, which is associated with poor survival.^21^

Although escalated ventilation parameters were determined as predictors for mortality before and are part of the RESP and PRESERVE scoring system, the FiO_2_ after ECMO initiation was never among those parameters.^17,22^ Probably, the outcome of these patients is not only influenced by ventilation parameters, but also by FiO_2_ and sweep gas flow of the ECMO system, which were not scrutinized in our study.

LDH is an important enzyme of the anaerobic metabolic pathway and presents a marker for bad end-organ perfusion. Up to date, it has only been described as prognostic marker for ARDS in COVID-19 patients receiving ECMO.^23^ In our cohort, only 20 of the deceased VV ECMO patients suffered from ARDS due to COVID-19, which makes a causality between LDH and COVID-19 unlikely.

An elevated INR is known to be a predictor for bleeding complications, especially intracranial hemorrage, in VV ECMO patients.^24^ While elevated INR 1 day after ECMO initiation had a negative impact on survival, there is no causality with bleeding complications, as only 4% of our VV ECMO patients suffered death due to bleeding. However, although there is no evidence in the literature for an elevated INR as a predictive factor for death on extracorporeal support, Esper et al. found a connection between elevated peak INR levels and 90-days and 1-year mortality in VA and VV ECMO patients.^25^

### Limitations

The limitations of our study lie in the retrospective single-center design of the study, which makes room for potential biases. We examined our patient cohort over 15 years, a long period where shifts in practice and ECMO management took place, that we didn’t consider in our analysis.

As our research represents a single-center experience, results may not be generalizable to other institutions.

## Conclusion

Despite the increasing use and advance of ECMO therapy, overall mortality remains high with 37.6% of patients dying while still on support. In VA ECMO patients, CPR and acidosis pre-implantation were found to be risk factors for dying on support, while the need of high doses of inotropic agents and high FiO2, elevated LDH and INR levels 1 day after ECMO initiation indicated poor prognosis in VV ECMO patients. Causes of death on VV ECMO were mainly due to refractory respiratory failure and sepsis, while VA ECMO patients died from cerebral ischemia, LCO and MOF with differences according to VA ECMO indication (ECPR: cerebral ischemia and LCO; postcardiotomy patients: LCO and MOF). To confirm these results, further studies are needed to identify possible predictors and patients with high risk of dying on ECMO to improve the outcome.
